# The Role of Transesophageal Echocardiogram in the Diagnosis and Treatment of Intracardiac Tumors: A Case of Atrial Myxoma

**DOI:** 10.7759/cureus.53597

**Published:** 2024-02-05

**Authors:** Filipa Côrte-Real, Hugo Côrte-Real

**Affiliations:** 1 Intensive Care Department, Hospital Central do Funchal, Funchal, PRT; 2 Intensive Care Department, Hospital Santa Maria, Lisbon, PRT

**Keywords:** cardiac neoplasm, transthoracic and transesophageal echocardiography, intensive care medicine, cardiothoracic & vascular surgery, large atrial myxoma

## Abstract

Cardiac myxoma is the most common primary heart tumor in adults. Although biologically benign, they can be life-threatening by obstructing heart function. They typically develop in the left atrium and can be polypoid (causing more obstruction) or papillary (more likely to cause embolizations). Symptoms are nonspecific, and diagnosis is relatively rare. Echocardiography is essential for quick diagnosis, and surgical removal is the primary treatment with low mortality rates, excellent postoperative survival, and low recurrence rates.

We report a 73-year-old woman presented to the emergency room with extreme fatigue and weight loss. Further investigations revealed a mass in the left atrium suggestive of an intracardiac tumor on a thoracic computer tomography scan. A subsequent transesophageal echocardiogram was performed, which showed a large, mobile, and friable hyperechogenic intra-auricular mass adhered to the atrial septum with moderate mitral regurgitation and moderate aortic stenosis. This case highlights the crucial role that the transesophageal echocardiogram plays in these patients by accelerating diagnosis, assisting with myxoma resolution, and confirming the complete removal of the myxoma.

## Introduction

A myxoma is a benign growth in the heart, that develops most frequently at the atrium, growing from the septum between the two atriums of the heart. Myxomas are rare, and the pathophysiology isn’t totally clear. They can be as small as a few millimeters or grow to a few centimeters. They occur more commonly in females and people aged 40 and above, and around 10% of myxomas are genetic [1,2]. Atrial myxomas are sometimes linked with valve obstruction stenosis and atrial fibrillation [2]. We describe a female patient with a huge atrium myxoma, that was discovered by accident, after a computer tomography scan (CT scan). She presented complaints of fatigue and weight loss, with a history of a localized right breast neoplasm. The patient was taken for removal of the mass and implantation of a biological mitral valve and was discharged without any symptoms.

## Case presentation

A 73-year-old woman presented to the emergency room (ER) with fatigue on great exertion without any cardiac symptoms, such as chest pain, palpitations, dyspnea, or syncope. She was accompanied by her son, who reports significant weight loss (about 8-10 kg) in the last 6 months (which they related to stress). There was a history of a localized right breast neoplasm, submitted to total mastectomy approximately 30 years ago, asthma and hypertension, and she was regularly medicated with olmesartan + amlodipina 20+5 mg and montelucaste 10 mg, with no history of drug allergies. On physical examination, a holosystolic murmur was noted throughout the precordium with radiation to the left axilla. No other relevant clinical signs were found.

She was admitted to the internal medicine ward where she was thoroughly investigated: electrocardiography and blood test with any relevant abnormalities but a CT pulmonary abdominal and pelvic showed a content inside the left atrium with suggestive semiology of intracardiac thrombus and no unequivocal evolutionary images attributable to pleuropulmonary, liver, abdominal, ganglion, or bone metastasis. Subsequently, she did a transesophageal echocardiogram that showed severe aortic stenosis and a hyperechogenic voluminous, very mobile, and friable intra-auricular mass adhering to the atrial septum with a moderate mitral regurgitation and a moderate aortic stenosis (Videos 1-2).

**Video 1:** Improvement of mitral regurgitation post-surgery

**Video 2:** Atrial myxoma before surgery

During hospitalization, she remained in functional class I, with no cardiovascular complaints. Given the suspicion of a left atrial thrombus, she started infusion of unfractionated heparin (UFH), and she performed an echocardiographic reassessment 5 days after initiation of UFH with no evidence of reduction in intra-auricular mass. A surgical proposal was sent to the cardiothoracic surgery team and was accepted.

The patient underwent surgery to remove the left atrium mass, which was sent to the pathologic anatomy, and implantation of an Intuity 23 biological valve in the aortic position. The intraoperative transesophageal echocardiogram confirmed the complete removal of the mass and resolution of mitral regurgitation (Videos 3-4).

**Video 3:** Atrial myxoma with mitral regurgitation

**Video 4:** Post-surgery of atrial myxoma

The patient remained in the cardiothoracic intensive care unit for three days, during which she required low-dose vasopressor support, which was quickly discontinued. Given the clinical picture, intraoperative transesophageal echocardiogram, and surgical findings a likely diagnosis of auricular myxoma was made, which was confirmed by pathologic anatomy. The patient was discharged without any symptoms and with an improvement in cardiac function.

## Discussion

A cardiac myxoma is the most common primary heart tumor in adults [1-3]. Although they are biologically benign, they can still be life-threatening if they affect heart function and can cause embolism for any part of the body [1]. These tumors typically develop in the left atrium (in 74%), like in our patient, but can also occur in the right atrium (about 18%), hence they are often known as atrial myxomas [2]. The rest form in the ventricles. A myxoma usually grows from the atrial septum and it may be attached by a pedicle, making it mobile. This mobility can lead to embolism or interference with heart flow if the tumor is large enough to obstruct a heart valve [3]. Our patient has a huge mobile myxoma that enters completely to the left ventricle during diastole, nevertheless, she didn’t have history of embolism neither syncope nor symptoms of low flow, the only symptom was fatigue. Myxomas can range in size, with some reaching up to 15 cm. They are more common in women between the ages of 30 and 60, although they can also be familial as a part of the Carney complex (usually diagnosis is made at the age of 20 and more common in men) [1-3]. There are two morphological forms of myxomas: polypoid and papillary, with polypoid forms more likely to cause obstruction and papillary forms more prone to embolization [1,2].

Clinical presentation is very unspecified, like lethargy, weight loss, shortness of breath, or fainting when standing (due to the obstruction of the mitral valve) [1-3]. Saying that the triad of intracardiac obstruction, embolic events, and constitutional symptoms should let us think about a myxoma [2]. Diagnosis can be made through imaging techniques such as echocardiography, cardiac magnetic resonance, and computed tomographic scanning, however, it can be mistaken for an intracardiac thrombus [3,4]. Therefore, patients are often treated with anticoagulation before repeating the exams to check for changes in thrombus, like in our patients. The definitive diagnosis is only done with histopathological examination [4]. Blood tests can reveal anemia, thrombocytopenia, and leukocytosis due to inflammation [4]. Surgical excision is the mainstay of treatment for myxomas, with low operative mortality, excellent postoperative survival, and low recurrence rate [1,2,4]. All this was done in the patient, who after excision of the myxoma there was a clear improvement in fatigue and mitral regurgitation.

## Conclusions

By presenting this clinical case, we aim to emphasize the significance of considering the diagnosis, as myxomas can either be asymptomatic or imitate various cardiovascular disorders. Consequently, physicians should be prepared to promptly diagnose and treat them with zeal to prevent potentially life-threatening complications.

This case highlights the crucial role that the transesophageal echocardiogram plays in these patients by accelerating diagnosis, assisting with myxoma resolution, and confirming the complete removal of the myxoma.
